# The Value of Combining Carbon Dioxide Gap and Oxygen-Derived Variables with Lactate Clearance in Predicting Mortality after Resuscitation of Septic Shock Patients

**DOI:** 10.1155/2021/6918940

**Published:** 2021-09-25

**Authors:** Walid Ahmed, Mohamed Laimoud

**Affiliations:** Critical Care Medicine Department, Cairo University, Cairo, Egypt

## Abstract

**Background:**

Achieving hemodynamic stabilization does not prevent progressive tissue hypoperfusion and organ dysfunction during resuscitation of septic shock patients. Many indicators have been proposed to judge the optimization of oxygen delivery to meet tissue oxygen consumption.

**Methods:**

A prospective observational study was conducted to evaluate and validate combining CO_2_ gap and oxygen-derived variables with lactate clearance during early hours of resuscitation of adults presenting with septic shock.

**Results:**

Our study included 456 adults with a mean age of 63.2 ± 6.9 years, with 71.9% being males. Respiratory and urinary infections were the origin of about 75% of sepsis. Mortality occurred in 164 (35.9%) patients. The APACHE II score was 18.2 ± 3.7 versus 34.3 ± 6.8 (*p* < 0.001), the initial SOFA score was 5.8 ± 3.1 versus 7.3 ± 1.4 (*p*=0.001), while the SOFA score after 48 hours was 4.2 ± 1.8 versus 9.4 ± 3.1 (*p* < 0.001) in the survivors and nonsurvivors, respectively. Hospital mortality was independently predicted by hyperlactatemia (OR: 2.47; 95% CI: 1.63–6.82, *p*=0.004), PvaCO_2_ gap (OR: 2.62; 95% CI: 1.28–6.74, *p*=0.026), PvaCO_2_/CavO_2_ ratio (OR: 2.16; 95% CI: 1.49–5.74, *p*=0.006), and increased SOFA score after 48 hours of admission (OR: 1.86; 95% CI: 1.36–8.13, *p*=0.02). A blood lactate cutoff of 40 mg/dl at the 6th hour of resuscitation (T6) had a 92.7% sensitivity and 75.3% specificity for predicting hospital mortality (AUROC = 0.902) with 81.6% accuracy. Combining the lactate cutoff of 40 mg/dl and PvaCO_2_/CavO_2_ ratio cutoff of 1.4 increased the specificity to 93.2% with a sensitivity of 75.6% in predicting mortality and with 86.8% accuracy. Combining the lactate cutoff of 40 mg/dl and PvaCO_2_ gap of 6 mmHg increased the sensitivity to 93% and increased the specificity to 98% in predicting mortality with 91% accuracy.

**Conclusion:**

Combining the carbon dioxide gap and arteriovenous oxygen difference with lactate clearance during early hours of resuscitation of septic shock patients helps to predict hospital mortality more accurately.

## 1. Background

Early detection of tissue hypoperfusion and rapid, efficient resuscitation is fundamental in the successful management of patients presenting with septic shock [1]. Recent guidelines from the Surviving Sepsis Campaign for management of septic shock patients focused on hemodynamic support through a systematic protocol of fluids and vasopressor therapy. The goal was to improve tissue perfusion and meet tissue oxygen demands [2]. The guidelines recommended continuing resuscitation and restoring mean arterial pressure (MAP) ≥ 65 mmHg with lactate clearance. This was based on the understanding that lactate clearance could serve as a surrogate for the reversal of global tissue hypoxia [3, 4]. Many indicators have been proposed to determine optimization of oxygen delivery (DO_2_) to meet tissue oxygen consumption (VO_2_) [5, 6]. However, achieving hemodynamic stabilization does not prevent progressive tissue hypoperfusion and multiorgan dysfunction [7]. No consistent advantages have been found for lactate guided resuscitation over using oxygen indicators [8–10]. The venoarterial carbon dioxide difference (PvaCO_2_) has been proposed as an indicator of tissue hypoperfusion [11–13]. A persistently high PvaCO_2_ gap despite resuscitation efforts may anticipate lactate changes and adverse outcomes [14] as CO_2_ production does not exceed O_2_ availability during aerobic conditions. Thus, the ratio between the PvaCO_2_ and the arteriovenous oxygen content difference (CavO_2_) may detect patients with anaerobic metabolism. Mekontso-Dessap et al. [15] reported that a PvaCO_2_ to CavO_2_ ratio of 1.4 was superior to PvaCO_2_ in predicting hyperlactatemia. The goal of this prospective observational study was to evaluate predictors of outcomes by combining the CO_2_ gap to CavO_2_, PvaCO_2_/CavO_2_ ratio, and lactate clearance during early hours of resuscitation of adult patients presenting with septic shock.

## 2. Methods

### 2.1. Patient Enrollment

This study was conducted at Cairo University Hospitals between January 2018 and February 2020 after getting the approval of the ethical committee. Informed consents were obtained from the enrolled patients. All 18-year-old or older consecutive patients who presented with septic shock were enrolled in the study. Septic shock was diagnosed as sepsis with persisting hypotension and a blood lactate level >2 mmol/L (18 mg/dl) despite adequate fluid resuscitation and necessitating vasopressors to get a mean arterial blood pressure ≥65 mm Hg [2]. Sepsis was defined as critical organ dysfunction due to a dysregulated body response to infection and associated with an acute change in Sequential Organ Failure Assessment (SOFA) score ≥ 2 [2].

Exclusion criteria included patients below 18 years of age, patients with chronic obstructive pulmonary disease or interstitial pulmonary diseases, refusal to consent, conditions that could affect lactate clearance such as chronic liver disease or alcoholism, diabetic patients on metformin therapy, and patients with different ventilation parameters during the 2 points of measurements.

### 2.2. Protocol of Resuscitation

All enrolled patients were resuscitated according to the Surviving Sepsis Campaign guidelines [2, 16]. Resuscitation started with crystalloids (30 ml/kg), and norepinephrine was started in the first hour as the first-choice vasopressor to maintain MAP ≥65 mm Hg. Cultures were obtained before starting broad-spectrum antibiotics. Central venous catheterization was performed via a jugular or subclavian approach, and the tip of the catheter was confirmed in the upper part of the right atrium by a chest X-ray. Arterial catheterization was performed via a radial or femoral approach, and the catheter position was confirmed by arterial waveform and blood gases analysis. Simultaneous arterial and central venous blood gases samples were obtained and analyzed.

### 2.3. Measured Variables

- Oxygenation and ventilation parameters were compared at the start of resuscitation (T0) and after 6 hours of resuscitation (T6) including arterial oxygen tension (PaO_2_), arterial carbon dioxide tension (PaCO_2_), arterial oxygen saturation (SaO_2_%), central venous oxygen tension (PcvO_2_), central venous carbon dioxide tension (PcvCO_2_), central venous oxygen saturation (ScvO_2_%), the arterial oxygen content (CaO_2_), and central venous oxygen content (CvO_2_). The difference between arterial and venous oxygen content (CavO_2_), oxygen extraction ratio (ER O_2_%), CO_2_ gap between venous and arterial samples (PvaCO_2_), and PvaCO_2_/CavO_2_ ratio were calculated as follows: ER O_2_% = (CaO_2_ − CvO_2_)/CaO_2_ and PvaCO_2_ = PvCO_2_ − PaCO_2_ [17].
- Lactate was recorded at 3 points: before (T0), after 6 hours (T6), and after 12 hours (T12) of resuscitation. Lactate clearance and delta changes were calculated from the difference between the second or the third reading and the first reading, divided by the first reading. This determined maximal change in the second or third reading.
- APACHE II and SOFA scores were calculated upon admission, as well as SOFA scores after 48 hours.
- Hospital mortality was the primary outcome, while the secondary outcomes included ICU stay and the need for hemodialysis.

### 2.4. Statistical Analysis

- Data are reported as mean ± standard deviation (±SD), median with interquartile range (IQR), or the number of cases and relative frequencies (percentages) when appropriate. Comparison of quantitative variables between the study groups was calculated using the *t*-test or Mann-Whitney *U* test for independent samples. For categorical data, a chi-square test was performed. Correlation between various variables was done using the Pearson correlation coefficient for continuous variables. A probability value (*p* value) less than 0.05 was considered statistically significant. Receiver operator characteristic (ROC) analysis was used to determine the optimum cutoff values for determining mortality.
- We developed the lactate-PvaCO_2_ score as follows: after multivariate logistic regression analysis to identify independent risk factors associated with mortality, the probability of using a stepwise analysis model was 0.05 for entry and 0.1 for removal. We tested various regression models using four variables (lactate, PvaCO_2_, CavO_2_, and PvaCO_2_/CavO_2_). We evaluated the final model (lactate-PvaCO_2_) for the goodness of fit using the Hosmer-Lemeshow test (*p* > 0.05). The variables were classified in the final model into clinically meaningful categories, and the estimated risk of mortality was recorded in each category. Binary logistic regression was run to explore potential predictors (following fluid resuscitation) for mortality. A score was calculated according to the regression output as follows: mortality probability=1/(1+exp(−(−8.968+(0.119*∗*lactate)+(0.379*∗*PvaCO_2_)))). “exp” is an exponential value (Tables 1 and 2).
- All statistical calculations were done using SPSS (Statistical Package for the Social Sciences; SPSS Inc., Chicago, IL, USA) version 26 for Microsoft Windows.

## 3. Results

### 3.1. Baseline and Clinical Characteristics of the Studied Patients

A total of 456 adult patients were included in the study with a mean age of 63.2 ± 6.9 years, with 71.9% being males. Respiratory and urinary infections were 75% of sepsis etiology. The nonsurvivors were significantly older (65.8 ± 7.2 versus 61.4 ± 4.6, *p*=0.001) with more frequent rates of chronic kidney disease (61.6% versus 40.1%, *p*=0.02) and less frequent rates of diabetes mellitus (60.4% versus 64.4%, *p*=0.04) compared to the survivors. Mortality occurred in 164 (35.9%) patients. The APACHE II score was 18.2 ± 3.7 versus 34.3 ± 6.8 (*p* < 0.001) and the initial SOFA score was 5.8 ± 3.1 versus 7.3 ± 1.4 (*p*=0.001), while SOFA score after 48 hours was 4.2 ± 1.8 versus 9.4 ± 3.1 (*p* < 0.001) in the survivors and nonsurvivors, respectively. The nonsurvivors had significant hypoalbuminemia (3.2 ± 1.6 versus 3.6 ± 1.3, *p*=0.02) and a higher mean serum creatinine level (1.6 ± 0.6 versus 1.2 ± 0.2, *p*=0.04) compared to the survivors. The survivors had a significantly longer length of ICU stay (6.8 ± 2.1 versus 3.6 ± 1.7, *p* < 0.001) but a lesser need for hemodialysis (10.3% versus 23.2%, *p*=0.02) compared to the nonsurvivors (Table 3).

### 3.2. The Studied Hemodynamic Variables

**Figure 1:**
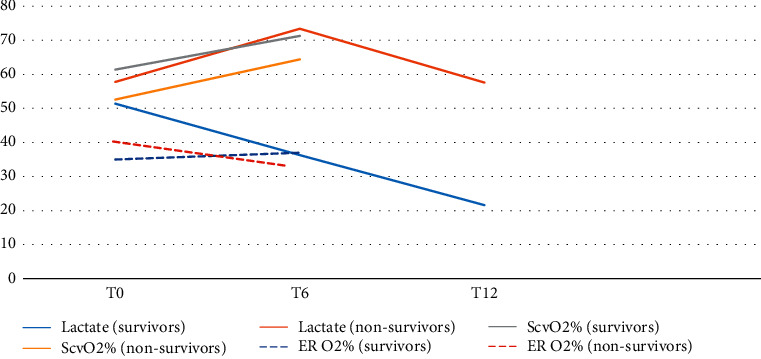
Oxygen extraction and blood lactate trends in the survivors and nonsurvivors.

The mean arterial blood pressure (MAP), heart rate, and temperature measurements were similar in both groups. Before starting resuscitation, the nonsurvivors had a significantly lower mean ScvO_2_% (52.6 ± 8.8 versus 61.4 ± 10.3, *p*=0.003) and PvaCO_2_/CavO_2_ ratio (1.8 ± 0.15 versus 2.2 ± 0.81, *p*=0.013) with a higher oxygen extraction ratio (40 ± 7.3% versus 35 ± 8.1%, *p*=0.002) compared to the survivors. However, there were no significant differences between both groups in blood lactate levels, arterial PO_2_, arterial oxygen content, arterial PCO_2_, or carbon dioxide gap (PvaCO_2_) at the initiation of resuscitation (Table 4 and Figure 1).

**Figure 2:**
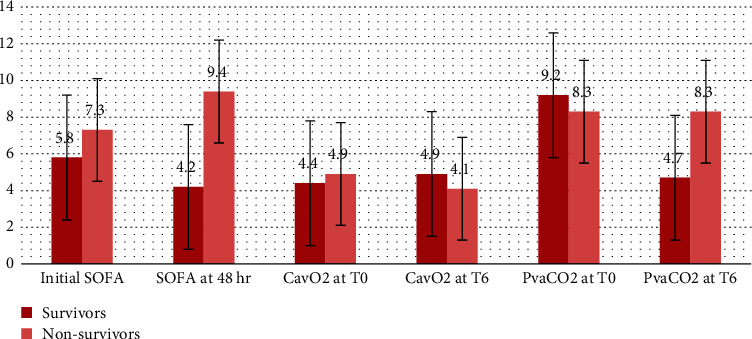
The SOFA score, CO_2_ gap, and oxygen content difference between the survivors and nonsurvivors.

After 6 hours of resuscitation, the survivors showed a significantly higher MAP (69.8 ± 5.4 versus 60.6 ± 4.8, *p* < 0.001), a lower mean blood lactate level (36.3 ± 14.4 versus 73.4 ± 30.4, *p* < 0.001), a higher arteriovenous oxygen content difference (4.9 ± 1.4 versus 4.1 ± 1.3, *p*=0.004), a lower carbon dioxide gap (4.7 ± 3.62 versus 8.3 ± 4.7, *p* < 0.001), a lower PvaCO_2_/CavO_2_ ratio (1.2 ± 0.72 versus 2.1 ± 1.13, *p* < 0.001), a higher ScvO_2_% (71.3 ± 11.2 versus 64.4 ± 8.4, *p*=0.038), and lesser doses of norepinephrine drip (0.28 ± 0.23 versus 1.02 ± 0.11, *p* < 0.001) compared to the nonsurvivors. After 12 hours of resuscitation, the mean blood lactate level was 21.6 ± 8.7 versus 57.6 ± 16.3 (*p* < 0.001 ) in the survivors and nonsurvivors, respectively (Table 4 and Figures 1 and 2).

Paired comparisons showed a declining SOFA score in the survivors and increasing trend in the nonsurvivors (−2.0 [−1.9 to −1.4] versus 3 [2.1–3.1], *p* < 0.001), respectively. Lactate clearance was evident in the survivors, while lactate level elevation occurred in the nonsurvivors. The delta lactate change was −29.4% [−35% to −26%] versus 26.9% [24%–41%] (*p* < 0.001) in the survivors and nonsurvivors, respectively. ∆CavO_2_ was 14% [12%–27%] versus −21% [−20% to −8%] (*p* < 0.001) and ∆PvaCO_2_ was −57% [−49 to −26%] versus −8% [−12% to 23%] (*p* < 0.001), while the ∆PvaCO_2_/CavO_2_ ratio was −71% [−54% to −28%] versus 7% [4%–51%] (*p* < 0.001) in the survivors and nonsurvivors, respectively (Table 5).

Norepinephrine dosage changes were positively correlated with delta changes of PvaCO_2_ and lactate and PvaCO_2_/CavO_2_ (*r*: 0.304, 0.728, and 0.386 and *p* < 0.001, 0.001 &<0.001, respectively). Arteriovenous oxygen content delta changes were negatively correlated with delta changes of PvaCO_2_, lactate, and PvaCO_2_/CavO_2_ (*r*: −0.322, −0.436, and −0.4 respectively; *p* < 0.001 for all). Multivariate regression analysis showed that the delta change in oxygen (A-V) content was the only significant predictor of delta change in lactate, that is, lactate clearance (−0.478, CI 95%: −0.232 to −0.828, *p* = 0.001) in a model that included delta changes in PvaCO_2_, CavO_2_, and PvaCO_2_/CavO_2_.

### 3.3. The Predictors of Hospital Mortality

In a multivariate regression analysis, hospital mortality was independently predicted by hyperlactatemia (OR: 2.47; 95% CI: 1.63–6.82, *p*=0.004), PvaCO_2_ gap (OR: 2.62; 95% CI: 1.28–6.74, *p*=0.026), PvaCO_2_/CavO_2_ ratio (OR: 2.16; 95% CI: 1.49–5.74, *p*=0.006), and increased SOFA score after 48 hours (OR: 1.86; 95% CI: 1.36–8.13, *p*=0.02) (Table 6).

**Figure 3:**
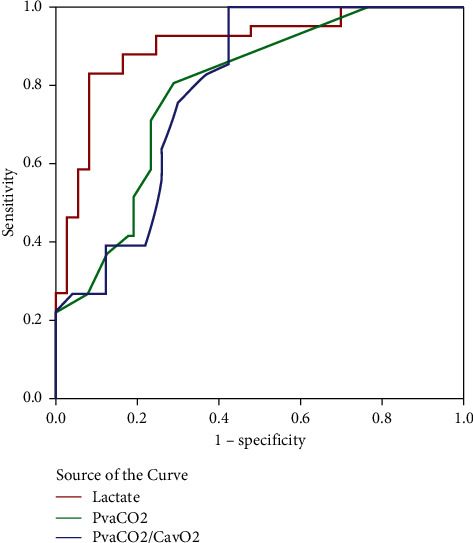
ROC analysis to determine possible cutoffs (after resuscitation) to predict in-hospital mortality. The blue line represents PvaCO_2_/CavO_2_. The green line represents PvaCO_2_. The red line represents lactate.

**Figure 4:**
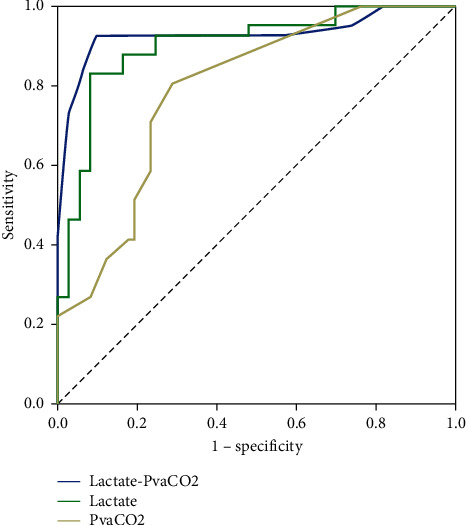
ROC analysis to determine possible cutoffs (after resuscitation) to predict in-hospital mortality. The blue line represents combined lactate and PvaCO_2_ curve. The green line represents lactate. The grey line represents PvaCO_2_.

The ROC analysis for cutoffs for the prediction of hospital mortality is summarized in Table 5. Blood lactate cutoff of 40 mg/dl at T6 had a 92.7% sensitivity and 75.3% specificity for predicting hospital mortality (AUROC = 0.902) with 67.9% PPV, 94.8% NPV, and 81.6% accuracy. A PvaCO_2_ cutoff of 6 mmHg at T6 had a 71% sensitivity and 77% specificity for predicting hospital mortality (AUROC = 0.791) with 63% PPV, 82%NPV, and 75% accuracy. PvaCO_2_/CavO_2_ cutoff of 1.4 at T6 had a 76% sensitivity and 70% specificity for predicting hospital mortality (AUROC = 0.793) with 58% PPV, 84% NPV, and 72% accuracy. Combining cutoffs for lactate (40 mg/dl) and PvaCO_2_/CavO_2_ ratio (1.4) increased the specificity to 93.2%, with a sensitivity of 75.6% in predicting mortality and 86.8% accuracy. Combining cutoffs for lactate (40 mg/dl) and PvaCO_2_ gap (6 mmHg) increased the sensitivity to 93% and increased the specificity to 98% in predicting mortality with 91% accuracy (Table 7 and Figures 3 and 4).

## 4. Discussion

Our study's main results are that septic shock patients who do not survive have worsening lactate levels with increased PvaCO_2_ and PvaCO_2_/CavO_2_ ratio, even after resuscitation. Mortality is predicted by hyperlactatemia, increasing PvaCO_2_ gap, incrementing PvaCO_2_/CavO_2_ ratio, and high SOFA score after 48 hours. Combining the PvaCO_2_ gap and PvaCO_2_/CavO_2_ ratio with lactate measurements can help in resuscitation and mortality prediction of septic shock patients. Many parameters are measured during septic shock resuscitation, including arterial and central venous pressures, urine output, cardiac output, blood lactate level, and central venous oxygen saturation (ScvO_2_%). The goal of a MAP at least 65 mmHg by the guidelines of Surviving Sepsis Campaign (SSC) has been challenged by many studies because targeting a predefined MAP by fluids and vasopressors did not lead to increased survival [18–20]. Hernandez et al. [21] reported that sepsis-induced hypotension without hyperlactatemia was associated with low mortality and less risks of organ dysfunction. Houwink et al. [22] reported the greater importance of first 24 hours' lactate over the MAP during septic shock resuscitation. They divided patients into 4 subgroups according to blood lactate cutoff of 2 mmol/L and MAP cutoff of 65 mmHg and reported lower mortality in the groups with low blood lactate regardless of MAP [22].

Our study showed significantly higher oxygen extraction and lower ScvO_2_% in the nonsurvivors compared to the survivors during early resuscitation without significant differences in arterial oxygen content (CaO_2_), arterial oxygen pressure (PaO_2_), or hemoglobin level in both groups. This could explain the increasing oxygen demands and failure of the aerobic metabolism with a subsequent anaerobic pathway activation and hyperlactatemia. After 6 hours of resuscitation, oxygen extraction increased and was associated with lactate clearance and improving markers of anaerobic metabolism in the survivors compared to the nonsurvivors.

ScvO_2_% was widely recommended targeting ≥70% during the first 6 hours of septic shock resuscitation [23–26] and ScvO_2_% less than 70% was predictive of mortality [23, 27, 28]. However, ScvO_2_% was unable to differentiate survivors from nonsurvivors in other studies [29–31].

Our study showed that both the survivors and nonsurvivors had similar initial lactate levels, but, after resuscitation, lactate clearance was evident in the survivors. Progressive hyperlactatemia has been associated with mortality and other negative clinical outcomes in septic and nonseptic critically ill patients [3, 4, 10, 29, 32–34]. Despite the proven beneficial role of lactate clearance in guiding resuscitation, bedside physicians have a challenge when faced with stable hemodynamics but high lactate levels as this reflects persistence of tissue hypoperfusion or slow time to clearance. High blood lactate levels may result in unnecessary fluids given that this may result in worse outcomes [35–37]. We report that the nonsurvivors received more fluids during the first 6 hours of resuscitation compared to the survivors. Boyd et al. [35] reported that more fluids transfused during the first 12 hours of septic shock resuscitation were linked to morbidity and mortality. Sadaka et al. [36] linked more fluid balance at 24 hours after resuscitation to increased mortality and documented increased mortality from 42 to 58% if there were more than 6 liters of positive fluid balance.

The PvaCO_2_ gap has been used as an important marker of tissue perfusion and cardiac output [11–14, 38, 39]. Its value persists in septic shock resuscitation, especially after getting MAP to at least 65 mmHg and normalization of ScvO_2_% [11, 12, 14]. Our results showed a significant decrease of the PvaCO_2_ gap in the survivors after 6 hours of resuscitation compared to the nonsurvivors without a significant difference between their initial values at T0. PvaCO_2_ gap was also a predictor of mortality in the multivariate regression analysis. Moreover, ROC analysis showed the PvaCO_2_ gap cutoff of 6 mmHg had an AUROC of 0.79 but a lesser predictive value compared to blood lactate in predicting mortality. Combining lactate with PvaCO_2_ gap increased the sensitivity and specificity with AUROC of 0.93 and accuracy of 91% in predicting mortality. Ospina-Tascón et al. [14] reported that a persistently high PvaCO_2_ gap could independently predict adverse outcomes and lactate changes regardless of other oxygen-derived variables. The PvaCO_2_ gap is a simple and attractive goal in resuscitation but it is a physiologically complex tool and should be studied with oxygen changes as it may be normal despite tissue hypoperfusion if associated with high blood flows preventing CO_2_ accumulation. Also the PvaCO_2_ gap may increase without tissue hypoperfusion in aerobic and anaerobic conditions due to the Haldane effect as the relation between the CO_2_ partial pressure and the CO_2_ content is affected by O_2_ saturation, pH differences, and hemoglobin changes [40].

As CO_2_ production does not exceed O_2_ production during aerobic metabolism, correcting the PvaCO_2_ gap by using the ratio of PvaCO_2_ to the arteriovenous oxygen content difference (CavO_2_) may detect patients with a risk of anaerobic metabolism [14, 40, 41]. Mekontso-Dessap et al. [15] reported that a PvaCO_2_ to CavO_2_ ratio of 1.4 was superior to PvaCO_2_ in predicting hyperlactatemia and also reported the agreement between PvaCO_2_/CavO_2_ ratio and blood lactate levels. Our study showed a difference in the PvaCO_2_/CavO_2_ ratio combined with lactate clearance in the survivors compared to the nonsurvivors who develop increased ratio and lactate elevation after 6 hours of resuscitation. Our results showed that PvaCO_2_/CavO_2_ ratio was a predictor of mortality in the multivariate regression analysis. The PvaCO_2_/CavO_2_ ratio cutoff of 1.4 had an AUROC of 0.793 in differentiating mortality. Our ROC analysis to determine possible cutoffs for lactate, PvaCO_2_, and PvaCO_2_/CavO_2_ to predict mortality showed that blood lactate had a superior performance differentiating mortality. However, combining the cutoffs for lactate and PvaCO_2_ or PvaCO_2_/CavO_2_ provided higher specificity (98% and 93.2%, respectively) than blood lactate levels alone (75.3%) with higher accuracy. Our results were consistent with Ospina-Tascón et al.'s [42] study that described lactate and PvaCO_2_/CavO_2_ ratio as independent predictors of mortality after 6 hours of resuscitation. Interestingly, Ospina-Tascón et al. [42] reported that, after getting the MAP ≥65 mmHg and ScvO_2_% ≥ 65% in most enrolled patients, 48% of the studied patients had a PvaCO_2_/CavO_2_ ratio ˃1 and 62% of patients had a blood lactate ˃2 mmol/L. Ospina-Tascón et al. [42] reported that patients with combined high lactate and PvaCO_2_/CavO_2_ ratio at T6 had the worst outcomes. Patients with normalized lactate level and PvaCO_2_/CavO_2_ ratio had the best outcomes, while patients with normalized lactate but still high PvaCO_2_/CavO_2_ ratio had similar unfavorable outcomes to those with high lactate levels with normalized PvaCO_2_/CavO_2_ ratio.

We used SOFA score to assess the clinical severity of the studied patients and the degree of multisystem dysfunction after resuscitation. The SOFA score has been previously used in different critically ill patients and is linked to mortality and outcomes [43–45]. Our results revealed that the nonsurvivors had higher initial SOFA and increasing scores trend after 48 hours of admission compared to the survivors. Also, the increasing trend of SOFA score was an independent predictor of mortality after resuscitation. Mesquida et al. [46] used SOFA scoring and reported increased SOFA trend and PvaCO_2_/CavO_2_ ratio in nonsurvivors after resuscitation. Ospina-Tascón et al. [42] divided the septic shock patients into four groups based on the blood lactate and PvaCO_2_/CavO_2_ ratio and reported the highest scores in the group with combined lactate ≥2 mmol/L and PvaCO_2_/CavO_2_ ratio ˃1 and the lowest scores in patients with low lactate ˂2 mmol/L and PvaCO_2_/CavO_2_ ratio ˂1.

Finally, our data showed that septic shock patients who were unlikely to survive persistently had worsening lactate levels with high PvaCO_2_ and PvaCO_2_/CavO_2_ ratios, despite resuscitation. Increased oxygen extraction was associated with increased lactate clearance and improving markers of anaerobic metabolism. Adding PvaCO_2_ gap and PvaCO_2_/CavO_2_ ratio to lactate measurements can increase the accuracy of mortality prediction.

## 5. Conclusion

Combining the carbon dioxide gap and arteriovenous oxygen difference with lactate clearance during the early hours of resuscitation of adult patients with septic shock helps to predict hospital mortality more accurately.

## Figures and Tables

**Table 1:** Lactate-PvaCO_2_ model for estimated risk of mortality with point scoring according to its individual subscores.

Variables	Range		Point score
Lactate	1	6	1
	7	12	3
	13	18	4
	19	24	6
	25	30	8
	31	36	10
	37	42	11
	43	48	13
	49	54	15
	55	60	16
	61	66	18
	67	72	20
	73	78	22
	79	84	23
	85	90	25
	91	96	27
	97	102	28
	103	108	30
	109	114	32
	115	120	34
	121	126	35
	127	132	37
	133	138	39
	139	144	40

PvaCO_2_	1	6	3
	7	12	9
	13	18	14
	19	24	20

**Table 2:** Estimated risk of mortality according to the summed point scores.

Summed point score	Estimated mortality (%)
1	0.0
2	0.0
3	0.0
4	0.1
5	0.1
6	0.2
7	0.2
8	0.4
9	0.5
10	0.8
11	1.2
12	1.9
13	2.8
14	4.2
15	6.2
16	9.1
17	13.2
18	18.8
19	26.0
20	34.8
21	44.7
22	55.1
23	65.0
24	73.8
25	81.0
26	86.6
27	90.8
28	93.7
29	95.8
30	97.2
31	98.1
32	98.7
33	99.2
34	99.5
35	99.6
36	99.8
37	99.8
38	99.9
39	99.9
40 or above	100.0

**Table 3:** Baseline characteristics of the study population.

The studied variables		All patients (*N* = 456)	Survivors (*N* = 292)	Nonsurvivors (*N* = 164)	*p* value
Age (years)		63.2 ± 6.9	61.4 ± 4.6	65.8 ± 7.2	0.001
Gender (male)		328 (71.9%)	216 (73.9%)	112 (68.3%)	0.34
Diabetes mellitus, *n* (%)		287 (62.9)	188 (64.4)	99 (60.4)	0.04
Chronic kidney disease, *n* (%)		218 (47.8)	117 (40.1)	101 (61.6)	0.02
Chronic heart failure, *n* (%)		127 (28)	83 (28.4)	44 (26.8)	0.34
Coronary artery disease, *n* (%)		141 (30.9)	89 (30.4)	52 (31.7)	0.42
Previous cerebrovascular stroke, *n* (%)		26 (5.7)	18 (6.2)	8 (4.9)	0.7
Left ventricle EF (%)		52.3 ± 6.8	51.6 ± 8.7	49.4 ± 4.8	0.31
Source of sepsis, *n* (%)	Pneumonia	227 (49.8)	154 (52.7)	73 (44.5)	0.38
	Urinary	113 (24.8)	64 (21.9)	49 (29.8)	
	Abdominal	82 (17.9)	59 (20.2)	23 (14.1)	
	Soft tissue	34 (7.5)	21 (7.2)	13 (9.7)	
APACHE II score		24.3 ± 9.7	18.2 ± 3.7	34.3 ± 6.8	<0.001
SOFA score at admission		6.4 ± 1.6	5.8 ± 3.1	7.3 ± 1.4	0.001
SOFA score after 48 hours		6.2 ± 3.6	4.2 ± 1.8	9.4 ± 3.1	<0.001
Need for dialysis		68 (14.9%)	30 (10.3%)	38 (23.2%)	0.02
Length of stay (days)		5.6 ± 2.4	6.8 ± 2.1	3.6 ± 1.7	<0.001
Hemoglobin (g/dL)		10.6 ± 2.3	10.9 ± 1.2	9.82 ± 2.3	0.51
Platelet count (×10^3^/ml)		134.2 ± 52.6	146.7 ± 38.3	128.6 ± 52.7	0.2
INR		1.3 ± 0.71	1.2 ± 0.62	1.1 ± 0.45	0.61
Creatinine (mg/dL)		1.4 ± 0.3	1.2 ± 0.2	1.6 ± 0.6	0.041
Bilirubin (mg/dL)		2.1 ± 1.2	1.8 ± 1.3	2.4 ± 1.6	0.13
Albumin (mg/dL)		3.6 ± 1.4	3.6 ± 1.3	3.2 ± 1.6	0.02
AST(U/L)		21.4 ± 2.6	20.9 ± 2.8	26.3 ± 3.4	0.015
ALT(U/L		26.8 ± 3.1	24.4 ± 2.7	27.4 ± 3.4	0.22

APACHE II: Acute Physiology and Chronic Health Evaluation II; SOFA: Sequential Organ Failure Assessment; INR: international normalized ratio; ALT: alanine transaminase; AST: aspartate transferase. Data are presented as mean (±SD) or *N* (%).

**Table 4:** Hemodynamic and blood gases' variables of the studied patients.

The studied variables	All patients	Survivors	Nonsurvivors	*p* value
Initial values (T0)				
MAP (mmHg)	42.3 ± 5.4	43.6 ± 6.7	41.4 ± 3.1	0.43
Heart rate (beats/min)	106 ± 26.3	102 ± 38.3	114 ± 29.7	0.19
Temperature (°C)	37.2 ± 1.7	37.1 ± 1.9	37 ± 1.87	0.32
Norepinephrine (*μ*g/kg/minute)	0.41 ± 0.28	0.36 ± 0.21	0.67 ± 0.08	<0.001
Blood lactate (mg/dl)	53.8 ± 18.9	51.4 ± 15.2	57.8 ± 18.7	0.12
PaO_2_ (mmHg)	61.4 ± 24.4	62.6 ± 12.3	60.2 ± 26.4	0.74
CaO_2_ (ml/dL)	12.6 ± 1.3	12.1 ± 1.8	11.8 ± 1.7	0.25
CvO_2_ (ml/dL)	7.6 ± 1.7	8.2 ± 1.4	7.1 ± 1.3	0.003
CavO_2_ (ml/dL)	4.3 ± 1.3	4.4 ± 1.6	4.9 ± 1.1	0.023
ScvO_2_ (%)	57.6 ± 9.6	61.4 ± 10.3	52.6 ± 8.8	0.003
ER O_2_ (%)	36 ± 8.2%	35 ± 8.1%	40 ± 7.3%	0.002
PaCO_2_ (mmHg)	46.3 ± 8.6	37.4 ± 7.6	38.4 ± 8.7	0.571
PvCO_2_ (mmHg)	38.4 ± 7.4	46.3 ± 8.4	46.4 ± 10.2	0.81
PvaCO_2_ (mmHg)	9.1 ± 3.2	9.2 ± 3.4	8.3 ± 2.8	0.32
PvaCO_2_/CavO_2_	2.1 ± 0.72	2.2 ± 0.81	1.8 ± 0.15	0.013

Follow-up values (T6)				
MAP (mmHg)	62.7 ± 12.4	69.8 ± 5.4	60.6 ± 4.8	<0.001
Norepinephrine (*μ*g/kg/minute)	0.82 ± 0.38	0.28 ± 0.23	1.02 ± 0.11	<0.001
Dopamine (*μ*g/kg/minute)	5.63 (3.32–13.4)	4.6 (3.1–9.41)	6.34 (5.47–13.42)	0.06
Fluid intake (liters)	3.84 (2.62–6.24)	2.73 (2.42–4.35)	3.26 (2.94–6.31)	0.03
Lactate T6 (mg/dl)	49.4 ± 24.7	36.3 ± 14.4	73.4 ± 30.4	<0.001
PaO_2_ (mmHg)	84.2 ± 38.4	89.7 ± 31.2	71.7 ± 29.4	0.044
CaO_2_ (ml/dL)	13.7 ± 1.8	13.9 ± 1.4	12.7 ± 1.61	0.42
CvO_2_ (ml/dL)	8.3 ± 1.6	8.2 ± 1.7	8.7 ± 1.5	0.103
CavO_2_ (ml/dL)	4.6 ± 1.4	4.9 ± 1.4	4.1 ± 1.3	0.004
ScvO_2_ (%)	65.8 ± 9.7	71.3 ± 11.2	64.4 ± 8.4	0.038
ER O_2_ (%)	36.4 ± 8.9%	37 ± 9.4%	32.7 ± 9.5%	0.004
PaCO_2_ (mmHg)	41.7 ± 5.8	35.6 ± 4.3	36.6 ± 7.3	0.42
PvCO_2_ (mmHg)	36.2 ± 6.2	40.5 ± 4.3	45.3 ± 6.12	0.002
PvaCO_2_ (mmHg)	6.2 ± 4.6	4.7 ± 3.62	8.3 ± 4.7	<0.001
PvaCO_2_/CavO_2_	1.4 ± 1.1	1.2 ± 0.72	2.1 ± 1.13	<0.001
Lactate T12 (mg/dl)	37.8 ± 13.2	21.6 ± 8.7	57.6 ± 16.3	<0.001

MAP: mean arterial pressure; PaO_2_: arterial oxygen tension; CaO_2_: arterial oxygen content; CvO_2_: central venous oxygen content; CavO_2_: arteriovenous oxygen content difference; ScvO_2_%: central venous oxygen saturation; ER O_2_: oxygen extraction ratio; PaCO_2_: arterial carbon dioxide tension; PvCO_2_: venous carbon dioxide tension; PvaCO_2_: CO_2_ gap.

**Table 5:** Changes of the studied variables with resuscitation.

The studied variables	Survivors	Nonsurvivors	*p*
SOFA score change	−2.0 (−1.9 to −1.4)	3 (2.1–3.1)	<0.001
Norepinephrine dose change	−0.06 (−01 to 0.2)	0.1 (0.01–0.11)	<0.001
∆lactate (%)	−29.4 (−35 to −26)	26.9 (24–41)	<0.001
ΔCavO_2_ (%)	14 (12% −27)	−21 (−20 to −8)	<0.001
ΔPvaCO_2_ (%)	−57 (−49 to −26)	−8 (−12 to 23)	<0.001
ΔPvaCO_2_/CavO_2_ (%)	−71 (−54 to −28)	7 (4–51)	<0.001

Data are presented as median and interquartile range (IQR). SOFA change = SOFA at 48 hours − admission SOFA. Norepinephrine dose change = dose at T6 − dose at T0. Lactate, CavO_2_, PvaCO_2_, PvaCO_2_/CavO_2_% change = T6 − T0/T0.

**Table 6:** Multivariate regression analysis for predicting in-hospital mortality.

The studied variables	Odds ratio	95% CI	*p* value
APACHE II	1.28	0.87–1.34	0.31
Delta SOFA after 48 hours	1.86	1.36–8.13	0.02
Lactate	2.47	1.63–6.82	0.004
PvaCO_2_/CavO_2_	2.16	1.49–5.74	0.006
PvaCO_2_	2.62	1.28–6.74	0.026

**Table 7:** ROC analysis of studied variables in predicting mortality.

ROC analysis	Lactate	PvaCO_2_	PvaCO_2_/CavO_2_	Combined lactate-PvaCO_2_
Cutoff	40	6	1.4	20.5
Area under the curve	0.902	0.791	0.793	0.930
Sensitivity	92.7%	71%	76%	93%
Specificity	75.3%	77%	70%	98%
PPV	67.9%	63%	58%	84%
NPP	94.8%	82%	84%	96%
Accuracy	81.6%	75%	72%	91%
LR+	3.8	3.0	2.5	9.7
LR−	0.1	0.4	0.3	0.1

ROC: receiver operating characteristics; PPV: positive predictive value; NPP: negative predictive value; LR: likelihood ratio.

## Data Availability

The data used in this study are available from the corresponding author upon request.

## Additional Points

A PvaCO_2_ gap is a simple tool, but its validity is debatable in high-cardiac-output septic patients. Our study was conducted before the COVID-19 pandemic, and we do not know if these results can apply to patients with COVID-19 presenting with septic shock. We planned to include the cardiac output and response during resuscitation but were unable to do so because of resource limitations. Most of the studied patients had echocardiographic assessments after resuscitation. So, we included only the systolic function of the left ventricle EF.
